# Lichen planus pemphigoides following COVID-19 infection

**DOI:** 10.1016/j.jdcr.2024.01.042

**Published:** 2024-03-26

**Authors:** Elisa S. Gallo, Yasmin Yaniv, Avital Baniel, Oren Katz, Tal Zeeli

**Affiliations:** a Department of Dermatology, Tel Aviv Sourasky Medical Center, Tel Aviv, Israel; b Department of Medicine & Surgery, Vita-Salute San Raffaele University, Milan, Italy

**Keywords:** COVID-19 post-infection, lichen planus pemphigoides (LPP)

## Introduction

Dermatologic consequences of COVID-19 infection have been reported. Herein, we add a case of lichen planus pemphigoides (LPP) manifesting 1 month after COVID-19 infection, and 2 weeks after resolution of viral symptoms. The patient denied any past medical history. This case demonstrates that LPP may be induced by a viral infection, specifically COVID-19.

## Case report

A 45-year-old previously healthy female, on no recent medications or supplements, presented with a 6-week history of a pruritic rash, which began 2 wk after recovering from COVID-19. Initially, the rash appeared prominently in her left versus right axilla only. Resolution occurred with a high-potency steroid cream. Two weeks later, the rash reappeared on her upper limbs and trunk, and responded to the same treatment. One week thereafter, the rash returned, covering her neck to bilateral feet. Morphology of the rash was consistently the same each time it appeared. Phototherapy was initiated, however after 3 sessions, the rash worsened and due to intractable pain, especially plantar pain inhibiting any pressure on her feet, she was admitted to the inpatient dermatology ward.

**Fig 1:**
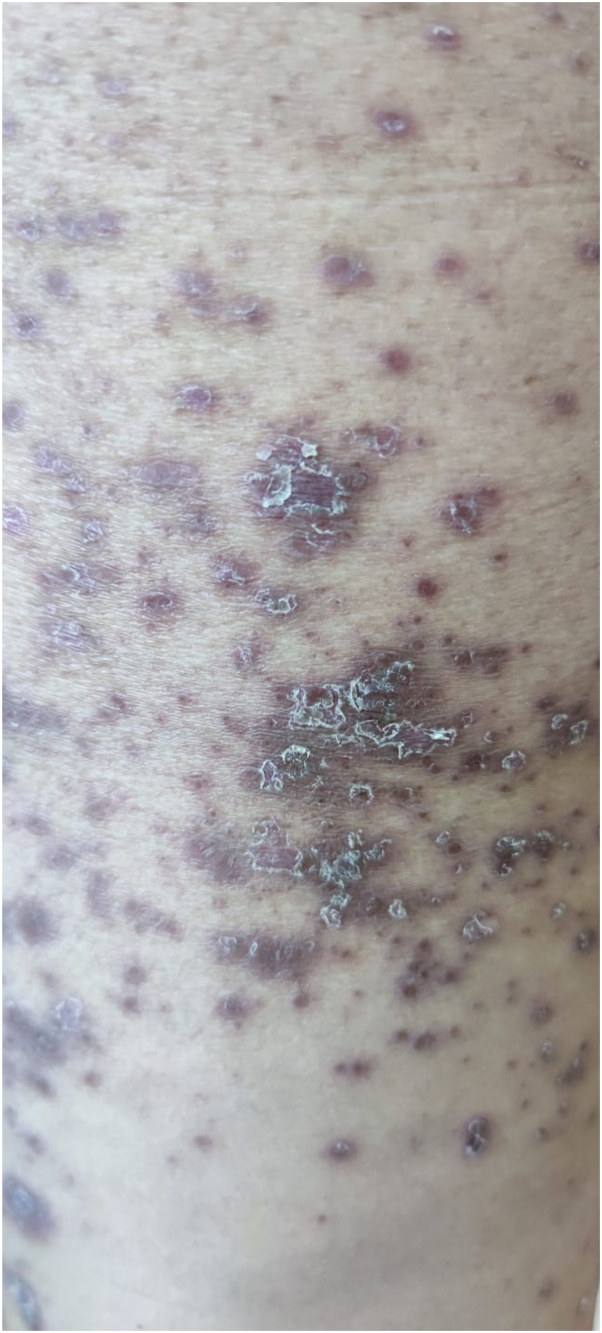
Lichen planus with scale.

**Fig 2:**
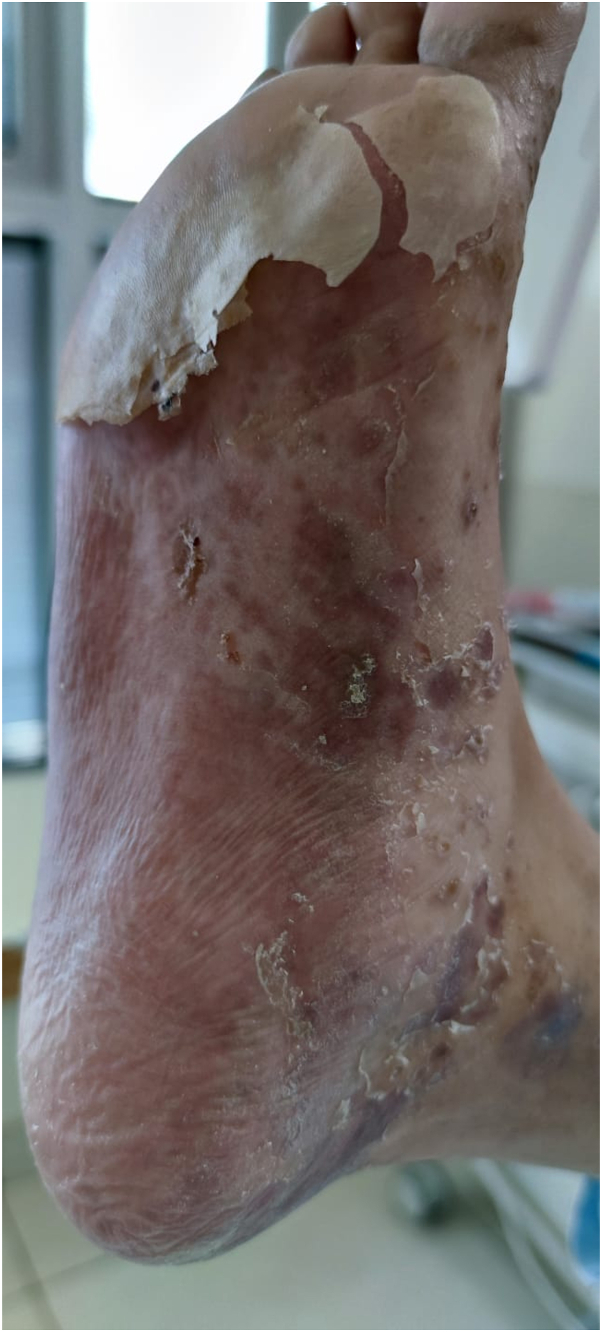
Desquamation following tense blister.

On examination, she had multiple purple polygonal papules and tense blisters diffusely from her neck to bilateral soles (Figs 1 and 2). Oral exam was positive for Wickham’s striae on the buccal mucosa. Complete blood count and metabolic panels were within normal limits. C-reactive protein was unremarkable (10 mg/dl). Biopsies were taken. Methylprednisolone 0.75 mg/kg IV daily × 4 days yielded a partial response only.

**Fig 3:**
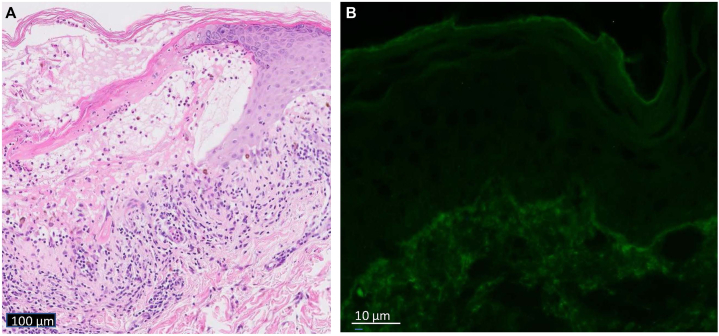
**A,** Histopathology revealing perivascular interface vacuolar subepidermal vesicular dermatitis with eosinophils, neutrophils, and melanophages. **B,** DIF positive for immunoglobulin G and C3 in a focal granular staining pattern at the dermal-epidermal junction and highlighted cytoid bodies. *DIF*, Direct immunofluorescence.

Pathology revealed perivascular interface vacuolar subepidermal vesicular dermatitis with eosinophils, neutrophils, and melanophages (Fig 3, *A*). Direct immunofluorescence was positive for immunoglobulin (Ig)G and C3 in a focal granular staining pattern at the dermal-epidermal junction and highlighted cytoid bodies; IgA and IgM were negative (Fig 3, *B*). Indirect immunofluorescence was negative. Salt-split skin was positive on the epidermal side. Bio-chip displayed fluorescence of the recombinant NC16a domain of BP180.

The patient was diagnosed with LPP. Given her pain and poor improvement, methylprednisolone was increased to 1 mg/kg intravenously, achieving disease control. Within days, full plantar desquamation occurred, and she tolerated weight-bearing activities.

2.5 weeks post discharge, after tapering her steroid (70 mg to 40 mg PO daily), the rash and pain worsened. As an outpatient, she continued prednisone 40 mg PO QD, and started oral methotrexate 5 mg the first week, which then was increased to 15 mg weekly with folate 5 mg orally twice weekly. 3 weeks later, no new blisters or papules appeared; she had diffuse postinflammatory hyperpigmented (PIH) macules on her back with residual purple polygonal papules on her upper and lower limbs. Remission was short-lived and new blisters and papules appeared. Her treatment regimen was changed to methotrexate 17.5 mg weekly, prednisone 5 mg daily, and folate 5 mg twice weekly. One year after LPP onset, she achieved a 3-month remission, with PIH remaining only. Following this remission, she experienced a flare of lichen planus, without blisters, and received acitretin to control flares and assist in PIH fading.

## Discussion

LPP is a rare autoimmune sub-epidermal blistering disease with an estimated prevalence of 1 per 1 million patients, an incidence in the fourth to fifth decade, and no sex predilection,^1^ versus a slight female preponderance.^2^ Although initially characterized as a type of bullous pemphigoid or lichen planus, evidence suggests it is a distinct disease.^3^ While lichenoid lesions are classic, LPP also displays autoantibodies against type XVII collagen (Col17, also known as BP180). Autoantibodies to Col17 are seen as well in bullous pemphigoid, pemphigoid gestationis, mucous membrane pemphigoid, and linear IgA dermatosis. However, LPP autoantibodies specifically target the non-collagenous NC16A linker domain of Col17, whereas bullous pemphigoid (BP) autoantibodies target NC17A1 through NC16A3.

First described by Kaposi in 1882 and coined, “Lichen Ruber Pemphigoides,”^4^ clinical findings of LPP include both lichenoid plaques and tense blisters and bullae. Although most often idiopathic, LPP has been associated with herbal supplements, angiotensin-converting enzyme inhibitors and hydroymethlglutaryl-CoA reductase inhibitors (statins),^3^ hepatitis B infection, and malignancy, specifically colonic adenocarcinoma.^1^ The diagnostic gold standard is direct immunofluorescence of perilesional skin, displaying autoantibody deposition along the dermal-epidermal junction, as first reported by Stingl and Hobular in 1975.^5^

The differential diagnosis of LPP includes bullous lichen planus (LP), wherein blisters form on pre-existing lichenoid papules and plaques. In contrast, LPP blisters are predominantly separate from LP lesions.^3^ While the latter scenario is typical, it is not absolute, as reports exist in which LPP blisters have been confined to LP lesions. Other factors for consideration include age of onset (LPP patients are younger than BP patients), location of lesions (LPP is seen mostly on the extremities – with more acral distribution of bullous lesions,^2^ whereas BP tends to be generalized), and the course of disease (LPP has a shorter, milder course than BP). Our patient conforms to 2 of these criteria, being middle-aged and experiencing significant bullous lesions acrally, though she has experienced a generalized distribution of LP lesions.

Due to the paucity of documented LPP cases, treatment guidelines are lacking. Systemic corticosteroids are accepted as first-line therapy, however despite subsequent LPP remission, there is a 20% recurrence rate.^1^ Second-line treatment includes hydroxychloroquine, mycophenolate mofetil, azathioprine, tetracyclines, nicotinamide, dapsone, isotretinoin, or ustekinamab.^6^ We chose methotrexate while systemic corticosteroids were tapered over several months, given that therapeutic success has been achieved in both LP and BP with methotrexate.

There are a number of reported dermatologic ramifications of COVID-19 infection including inflammatory exanthems manifesting as maculopapular, urticarial, or vesicular, to vascular findings such chilblain-like lesions (a.k.a. “COVID toes), and petechial, purpuric, or livedoid signs.^7^ Maculopapular and urticarial exanthems have tended to appear as initial manifestations of COVID-19 infection, whereas vesicular and vascular signs have occurred later. LPP has been reported following COVID-19 infection,^8^ and our case further supports this association. While an atypical polymorphic papulovesicular eruption was reported in middle-aged patients recovering from COVID-19, the diagnosis was not confirmed histopathologically.^9^ In another report, the need is emphasized for histopathologic analysis of dermatologic exanthems in the setting of post-COVID-19 infection.^10^ In our patient’s case, histopathology confirmed distinct features of LPP, which developed post-COVID-19 infection.

## Conflicts of interest

None disclosed.
